# Gastrostomy Tube Use after Transoral Robotic Surgery for Oropharyngeal Cancer

**DOI:** 10.1155/2013/190364

**Published:** 2013-07-08

**Authors:** Samer Al-khudari, Scott Bendix, Jamie Lindholm, Erin Simmerman, Francis Hall, Tamer Ghanem

**Affiliations:** ^1^Head & Neck Institute, Cleveland Clinic Foundation, Cleveland, OH 44106, USA; ^2^Wayne State University School of Medicine, Detroit, MI 48202, USA; ^3^Department of Otolaryngology-Head & Neck Surgery, Henry Ford Hospital, Detroit, MI 48202, USA; ^4^Division of Speech-Language Sciences and Disorders, Department of Neurology, Henry Ford Hospital, Detroit, MI 48202, USA

## Abstract

*Objective*. To evaluate factors that influence gastrostomy tube (g-tube) use after transoral robotic surgery (TORS) for oropharyngeal (OP) cancer. *Study Design/Methods*. Retrospective review of TORS patients with OP cancer. G-tube presence was recorded before and after surgery at followup. Kaplan-Meier and Cox hazards model evaluated effects of early (T1 and T2) and advanced (T3, T4) disease, adjuvant therapy, and free flap reconstruction on g-tube use. *Results*. Sixteen patients had tonsillar cancer and 13 tongue base cancer. Of 22 patients who underwent TORS as primary therapy, 17 had T1 T2 stage and five T3 T4 stage. Seven underwent salvage therapy (four T1 T2 and three T3 T4). Nine underwent robotic-assisted inset free flap reconstruction. Seventeen received adjuvant therapy. Four groups were compared: primary early disease (PED) T1 and T2 tumors, primary early disease with adjunctive therapy (PEDAT), primary advanced disease (PAD) T3 and T4 tumors, and salvage therapy. Within the first year of treatment, 0% PED, 44% PEDAT, 40% PAD, and 57% salvage patients required a g-tube. Fourteen patients had a temporary nasoenteric tube (48.3%) postoperatively, and 10 required a g-tube (34.5%) within the first year. Four of 22 (18.2%) with TORS as primary treatment were g-tube dependent at one year and had received adjuvant therapy. *Conclusion*. PED can be managed without a g-tube after TORS. Similar feeding tube rates were found for PEDAT and PAD patients. Salvage patients have a high rate of g-tube need after TORS.

## 1. Introduction

Oropharyngeal squamous cell carcinoma (OPSCCA) is the ninth most common type of cancer in the United States with an estimated 40,000 newly diagnosed this year. More than 7,000 of those diagnosed will die from OPSCCA [1]. An increased incidence of OPSCCA has been noted due to the human papilloma virus (HPV) [2]. Interestingly, HPV-related OPSCCA occurs in younger patients who are often nonsmokers and nondrinkers. Treatment of OSCCA in this cohort aims to reduce the morbidity of traditional radiation and chemoradiation regimens, which often have profound toxicity-related deglutition which can occur acutely and many years after treatment. 

Minimally invasive surgery has been increasingly utilized in treating oropharyngeal cancer, especially with the availability of transoral robotic surgery (TORS). This approach allows for focused treatment to the primary tumor tissue while minimally disrupting surrounding functional tissue of the oropharynx and avoiding morbidity associated with traditional open approaches to the oropharynx. Gastrostomy tube (g-tube) placement rates after TORS have been reported to range from 19% to 39.5% [3–5], with approximately 4% of patients using a feeding tube at one year [6]. The purpose of this study aimed to assess g-tube use in a cohort of OPSCCA patients undergoing TORS for early stage (T1 and T2), advanced stage (T3 and T4) disease, and salvage surgery.

## 2. Methods

Institutional Review Board approval was obtained for a retrospective review between March 2010 and July 2012 of head and neck cancer patients considered appropriate for robotic surgery at our urban tertiary care center. All patients were evaluated preoperatively and considered good surgical candidates for TORS based on anatomy and location of the tumors. Excluded patients included those with oral tongue, nasopharynx, supraglottis, and hypopharynx tumor locations. All remaining patients considered were either tonsil or base of tongue cancers. Squamous cell carcinoma pathology was present in all cases. All patients underwent TORS resection of the primary cancer with concurrent neck dissections as indicated by preoperative evaluation after discussion of care at a multidisciplinary tumor board. 

Patient outcomes recorded included the need for g-tube, tracheostomy, and free flap reconstruction. G-tube use was recorded before and after surgery and on serial followup when undergoing adjuvant therapy if required. Time points for evaluation of g-tube presence were 1, 3, 6, and 12 months postoperatively. Most patients were fed orally postoperatively, and those not able to tolerate oral feedings were fed via nasogastric tube for up to 21 days. Past this period, a g-tube was utilized if the patient still required nutritional support. Demographic and clinical information recorded included patient age, sex, race, tumor site and staging, and prior treatment. Operative data recorded included type of resection and histologic findings. Postoperative data included complications, length of hospital stay, adjuvant therapy received, and feeding tube status. The need for adjuvant therapy was determined by the tumor board after surgery was completed and staging was final. Free flap reconstruction was performed by the attending surgeon depending on the resultant surgical defect, usually after large resections or in patients with prior chemoradiation therapy to the region or for protection of the great vessels.

Once identified, patients were divided into two groups: primary treatment and salvage treatment. Salvage treatment patients had received prior treatment for head and neck cancer with either chemoradiation therapy or surgical resection. Further division separated the primary treatment group by T-stage and whether adjuvant therapy was given. Four groups were then compared: primary early disease (PED) T1 and T2 tumors, primary early disease with adjunctive therapy (PEDAT), primary advanced disease (PAD) T3 and T4 tumors, and salvage therapy. 

Kaplan-Meier and Cox hazards model evaluated effects of early (T1 and T2) and advanced (T3 and T4) disease, adjuvant therapy (chemotherapy and/or radiation), and free flap reconstruction on g-tube use. Last followup was recorded on all patients and ranged from 12 to 28 months.

## 3. Results

Twenty-nine patients met study criteria and were included in the data analysis. All patients had concurrent neck dissections as indicated clinically. Patient demographics (Table 1) included 24 men (82.8%) and five women (17.2%) with a mean age of 60.2 years (range 46–83 years). Twenty-two patients underwent TORS as primary treatment, 17 for early, and five for advanced disease. Of the seven patients who underwent salvage treatment, five received prior chemoradiation therapy, one prior surgery and chemoradiation therapy, and one prior radiation therapy only. Tumor location included 16 tonsil (55%) and 13 base of tongue (45%). Final pathology results yielded histologically all squamous cell carcinoma, with subtypes as follows: 19 squamous cell (65.5%), five nonkeratinizing squamous cell (17.2%), four basaloid squamous cell (13.8%), and one sarcomatoid (3.4%). Seventeen patients received adjuvant therapy (58.6%), either radiation therapy or a combination of chemotherapy and radiation therapy. Nine patients underwent robotic-assisted inset free flap reconstruction (31%), seven radial forearm free flaps (24.1%), one radial forearm free flap plus pectoralis major flap (3.4%), and one anterior lateral thigh free flap (3.4%). 

Average length of hospital stay was 4.3 days (range 1–11 days). Five patients required tracheostomy with an average duration of 42.8 days (range 8–150 days). Two patients required intubation postoperatively with an average duration of 24 hours. Fourteen patients had temporary nasoenteric Dobhoff tube (48.3%) postoperatively, and 10 patients required a g-tube (34.5%) during their treatment within the first year.

**Figure 1:**
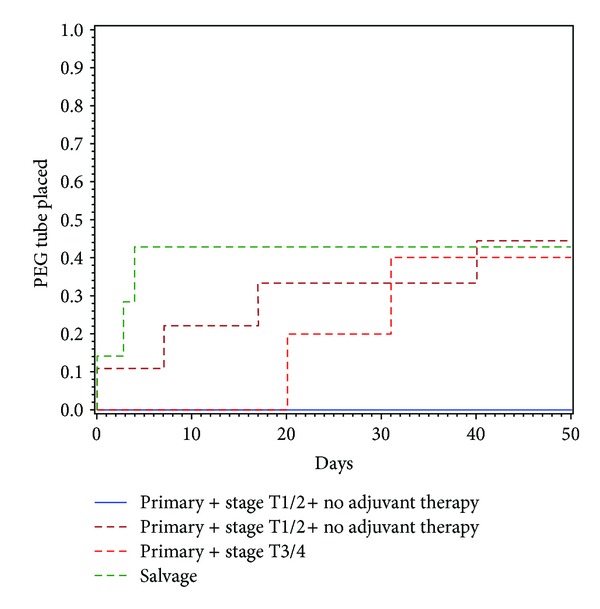
Kaplan-Meir analysis of g-tube placement.

T-stage breakdown consisted of seven T1, 14 T2, five T3, and three T4. Of the 22 primary treatment patients, eight were early stage T1 T2 without adjuvant therapy (PED), nine were early stage T1 T2 with adjuvant therapy (PEDAT), and five were late stage T3 T4 with adjuvant therapy (PAD). In the PED group, no patients required g-tube (0%). Four patients in the PEDAT group required g-tube (44.4%), and only two were g-tube dependent at 12-month followup (22.2%). Two patients in the PAD group required g-tube (40%), and two patients were g-tube dependent at 12-month followup (40%).  Table 2 shows overall g-tube rates and at 12-month followup. Four of the seven salvage patients required g-tube (57.1%). Two salvage patients remained g-tube dependent at 12-month followup (28.6%). Kaplan-Meier analysis comparison between the four groups showed no significant difference (*P* = 0.166) (Table 3, Figure 1). Four of five patients who underwent primary TORS treatment and reconstructed with a free flap required a g-tube, and two of four patients undergoing salvage therapy with a free flap required a g-tube (*P* = 0.99). Overall, four of nine (44.4%) patients who underwent free flap reconstruction were feeding tube dependent at one year.

## 4. Discussion

 Transoral surgery for OPSCCA, whether with transoral laser microsurgery (TLM) or TORS, may result in lower g-tube dependence than primary chemoradiation therapy and traditional open approaches [7]. Unfortunately, g-tubes may be necessary for some patients undergoing treatment of oropharyngeal cancer whether they are treated surgically or nonsurgically. Approximately 50%–70% of patients undergoing primary chemoradiation therapy may have severely impaired swallowing either during or after treatment and may require nutritional support via a feeding tube. Patient, tumor, and treatment factors all have been shown to impact the need for a feeding tube [7]. Rates of g-tube dependence after primary radiotherapy for OPSCCA have been reported to range from 15% to 25%, increasing to 18.1%–51% following chemoradiation. Published g-tube dependency rates following TLM with adjuvant therapy for OP cancers range from 0% to 18% [8]. The destructive effects of the tumor itself, surgery, radiation, and/or chemotherapy destroy the integrity of the surrounding structures and lead to impaired or even impossible oral intake after treatment, necessitating a g-tube.

Our study is unique given the high number of salvage patients, advanced T-stage disease, as well as the number of patients reconstructed with free flaps. No patients with early disease without adjuvant therapy required feeding tubes. Early stage tumors require less surgical resection and cause less surrounding tissue damage, which may translate to reduced need for a g-tube. Genden et al. recently reported 0% g-tube rate for early stage disease (T1 and T2) treated with primary TORS [9]. In a series of 38 (33 patients with T1 T2 tumors) with oropharyngeal carcinoma, Weinstein et al. reported an overall g-tube rate of 39.5% with only 2.7% g-tube dependent at 12 months [6]. Our results for early T-stage disease show comparable g-tube rates at 0% for those not requiring adjuvant treatment, 23.5% overall, and 11.8% dependent on g-tube at 12 months. In a series of 84 patients from Washington University undergoing TLM with adjuvant therapy as indicated with both early and advanced T-stage tumors, 46% of patients received a g-tube during treatment, but only 18.8% maintained a feeding tube at one year and 9.3% at two years. The largest number of feeding tubes was present at three months after TLM, which may be related to the adjuvant treatment [7]. Our data as well as results from other transoral surgical approaches suggests that patients with primary early disease not requiring adjuvant therapy can successfully return to oral diet without the need for g-tube placement during their treatment course. 

We have evaluated TORS to treat patients with advanced T-stage OPSCCA, and our g-tube use data was surprisingly similar for early T-stage disease requiring adjuvant treatment. We chose to evaluate our patients by the T-stage of the primary tumor because T stage has been shown to be an independent predictor of g-tube use in patients undergoing chemoradiation therapy. T stage or the size of the primary site also is an important consideration when performing transoral surgery of the oropharynx. When comparing patients treated for T1 T2 tumors with adjuvant therapy to those with T3 T4 with adjuvant therapy, there was no significant difference in g-tube need. Patients with larger primary cancers are more likely to require g-tubes, and this was not significantly higher than those with early T-stage primary patients with adjuvant therapy. We believe that this finding supports the notion that larger resections alone do not always result in increased g-tube use, especially if reconstruction is utilized. Instead, the need for adjuvant radiation therapy may affect the need for g-tube placement more so than T-stage, which might be due to the additive of effects of trimodality treatment to the oropharynx.

Patients undergoing salvage therapy for OP cancer have increased need for a g-tube when compared to patients with primary disease [10]. Patients undergoing prior therapy are likely to have preexisting impairment of the swallowing mechanism and therefore more likely to require a g-tube after further surgical treatment to the oropharynx. In our patient cohort, four of seven salvage patients required a g-tube during treatment and two of seven were g-tube dependent at one year. In a cohort of seven salvage patients treated with TORS at the University of Alabama, no patients were g-tube dependent at six months [11]. Further study of salvage treatment is needed, but clearly salvage oropharyngeal cancer can successfully be resected with TORS with relatively low g-tube dependence rate.

Smaller oropharyngeal cancers can be left to heal secondarily or by primary closure; however, larger defects after TORS may require reconstruction. Free tissue transfer with the robotic assistance for flap inset provides additional tissue and carotid coverage with avoidance of the standard lip-split, mandibulotomy approach, and improved functional results after large oropharyngeal resections [12]. Two studies described the utility of free tissue transfer in oropharyngeal reconstruction [9, 12]. In our series, 80% of primary TORS patients with free flaps required g-tube while 50% of salvage TORS received a g-tube. No significant difference in g-tube rates occurred between primary and salvage patients undergoing free flap reconstruction. At 12 months, four of nine patients (44.4%) who underwent free flap reconstruction required a feeding tube. We postulate that those patients requiring free flap reconstruction tend to have more advanced disease necessitating adjuvant therapy and thus may have multifactorial explanations why free flap reconstruction alone did not predict g-tube need. 

Some limitations of this study are its retrospective nature and nonrandomized approach. We did not assess the details of adjuvant treatment given the relatively smaller number of patients and various combinations of chemotherapy and radiation dosing postoperatively. Also some patients might have received a g-tube for prophylactic rather than reactive reasons during treatment. Nonetheless, this information is helpful to counsel patients on the overall rates of placement and dependence on a feeding tube when patients choose to undergo transoral robotic surgery and may or may not require adjuvant treatment.

With increasing incidence of OPSCCA, it is important to consider not only disease control with the various treatment options but also the toxicity and morbidity of the various treatments. While traditional surgery for OPSCCA was viewed negatively because of its invasive, highly morbid nature, transoral approaches allow for excellent oncological results with decreased morbidity. Current results indicate that further investigation into postsurgical adjuvant treatment doses may improve functional outcomes of patients while maintaining excellent disease control rates. As randomized trials are planned for surgical treatment of the oropharynx, the role of TORS will be better defined in OPSCCA.

## 5. Conclusion

Primary early oropharyngeal cancer can be managed without a g-tube after TORS. Similar g-tube rates were found for PEDAT and AD patients. Salvage patients have a high rate of g-tube use after TORS. This information is helpful when counseling patients on the potential need for a g-tube in the treatment course of their OP cancer.

## Figures and Tables

**Table 1:** Demographics and results.

	All patients	Primary treatment	Salvage treatment
	*N* = 29	*N* = 22	*N* = 7
Gender	M = 24 F = 5	M = 18 F = 4	M = 6 F = 1
Base of tongue	13	8	5
Tonsil	16	14	2
T stage			
T1 or T2	21	17	4
T3 or T4	8	5	3
Adjuvant chemotherapy and/or radiation	17	14	3
Free flap reconstruction	9	5	4

**Table 2:** Gastrostomy tube presence by disease status.

Group	Tumor	Overall G-tube %	12 Month G-tube %
1	Primary T1/2	0 (0/8)	0 (0/8)
2	Primary T1/2 + adjuvant therapy	44.4 (4/9)	22.2 (2/9)
3	Primary T3/4 + adjuvant therapy	40 (2/5)	40 (2/5)
4	Salvage treatment	57.1 (4/7)	28.6 (2/7)

**Table 3:** Log rank comparison of all 4 groups.

Comparison	*P*-value
All groups	0.166
T1 T2 versus T1 T2 + adj	*0.037 *
T1 T2 versus T3 T4 + adj	0.058
T1 T2 versus Salvage	*0.015 *
T1 T2 + adj versus T3 T4 + adj	0.771
